# Diagnostic utility of lung ultrasound in predicting the need for surfactant therapy in preterm neonates with respiratory distress

**DOI:** 10.3389/fped.2023.1307761

**Published:** 2023-12-04

**Authors:** Pari Singh, Suprabha Patnaik, Arjun Verma, Reema Garegrat, Rajesh Maheshwari, Pradeep Suryawanshi

**Affiliations:** ^1^ Neonatology, Bharati Vidyapeeth (Deemed to be) University Medical College, Pune, India; ^2^ Neonatology, Westmead Hospital, Westmead, NSW, Australia

**Keywords:** lung ultrasound score, prematurity, neonate, respiratory distress, surfactant therapy

## Abstract

**Background:**

Lung ultrasound is an accurate and early predictor for surfactant replacement therapy in respiratory distress syndrome (RDS) as compared to clinical parameters and chest x-ray. However, lung pathologies for respiratory distress at birth have overlapping symptomatology and low middle-income countries have a higher incidence of congenital pneumonia, in addition to RDS, making the immediate diagnosis difficult. Thus, there is a need for assessing a cutoff for lung ultrasound scores in the given setting.

**Objectives:**

The primary objective was to determine the diagnostic accuracy of the lung ultrasound score (LUS) in predicting the need for surfactant therapy in preterm neonates with respiratory distress. Secondary objectives were to correlate LUS with corresponding oxygen saturation to the fraction of inspired oxygen ratio (SpO2/FiO2), arterial/Alveolar oxygen pressure ratio (a/A), and chest x-ray (CXR) findings.

**Methodology:**

A prospective observational study was carried out at a tertiary-level neonatal intensive care unit in India in 2022 enrolling 100 neonates <34 weeks gestational age with respiratory distress at birth. After initial stabilization of the neonate, LUS was performed and baseline parameters were noted. Surfactant was administered as per the 2019 European Consensus guidelines and LUS was repeated after 6 h of therapy.

**Results:**

The mean gestation of enrolled neonates was 31.06 ± 2.12 weeks and the mean birthweight was 1,412 ± 391 g. Approximately 58% were diagnosed with RDS and 30% had congenital pneumonia. Surfactant was administered to 40% of neonates. The cutoff LUS for surfactant therapy was 7 [area under the curve (AUC) 0.977; 95% CI, 0.947–1; *P* < 0.001; with sensitivity 92.5%, specificity 96.67%, PPV 94.87%, and NPV 95.08%] and the cutoff LUS for the second dose of surfactant was 10 (AUC 0.964; 95% CI, 0.913–1; *P* < 0.001). The score decreased by 3.24 (2.44–4.05) after 6 h of the first dose and correlated significantly with SpO2/FiO2 ratio (−0.750), a/A ratio (−0.650), and CXR findings (0.801).

**Conclusion:**

The study predicted an optimal LUS cutoff of 7 and 10 for the need for the first dose of surfactant and re-treatment, respectively, in neonates <34 weeks gestational age with respiratory distress.

## Introduction

One of the most important causes of morbidity and mortality in preterm neonates, respiratory distress occurs in almost 5.8% of all live births (1) and accounts for 30%–40% of admissions to neonatal units (2). The management is based on earlier and timely administration of surfactant along with continuous positive airway pressure (CPAP) support. However, identifying the narrow window for administering surfactant remains challenging, given the advantages of early rescue surfactant therapy in reducing the risk of acute pulmonary injury, neonatal mortality, and chronic lung disease (3).

In recent times, lung ultrasound has gained a crucial role in the early diagnosis and evaluation of respiratory distress in neonates (4). It is a quick and safe bedside technique and can be repeated several times a day (5).

Over the years, studies have developed a lung ultrasound score (LUS) for the evaluation of lung aeration and prediction of surfactant administration (6, 7). The same was validated in 2015, demonstrating its utility in the management of respiratory distress syndrome (RDS) (8).

The indication of surfactant administration is variable among different neonatal intensive care units (NICU) and is guided by parameters such as chest x-ray (CXR), arterial/Alveolar oxygen pressure ratio (a/A), and FiO2. Presently, the guidelines for surfactant therapy are based on the fraction of inspired oxygen (FiO2) values (9), which may be arbitrary and non-specific (10).

In previous studies, lung ultrasound aided in guiding early rescue therapy within 2 h of delivery, thereby reducing the need for a repeat dose of surfactant, the need for invasive ventilation, and the number of days on a ventilator (11–14). When compared with chest x-ray, it was found to have higher specificity and sensitivity in predicting the need for surfactant in RDS (12).

Although the utility of LUS in predicting the disease severity and need for surfactant has been reported (15), data related to lung ultrasound scoring and its accuracy in predicting the need for surfactant in preterms with respiratory distress is lacking for the Indian population. It has the potential to be an accurate and early predictor of the need for surfactant therapy in this population cohort, thus, allowing timely treatment and reduced radiation exposure.

Therefore, this study was conducted to determine the diagnostic accuracy of LUS in predicting the need for surfactant in preterm neonates with respiratory distress and correlate it with corresponding oxygen saturation to the fraction of inspired oxygen ratio (SpO2/FiO2), a/A ratio, and findings on CXRs.

## Methodology

A single-centre prospective observational study was conducted in a tertiary care NICU attached to a medical college with approximately 1,500 annual admissions and 130–150 admissions per month. The study was approved by the institutional ethics committee and registered in the clinical trials registry. All inborn as well as outborn neonates <34 weeks gestational age admitted to the NICU within 24 h of life with respiratory distress were included. Informed consent was obtained from the patient's parents. Neonates with complex cyanotic congenital heart disease, congenital malformation, chromosomal abnormality, inborn error of metabolism, and congenital lung disorder were excluded. The included neonates were started on non-invasive respiratory support from the time of admission to the NICU with an appropriately sized nasal interface. Peak end-expiratory pressure (PEEP) was set at 6 cm H2O and FiO2 levels were adjusted to maintain target oxygen saturations within the 90% to 95% target range. In case of failure of CPAP support (not maintaining target oxygen saturation on PEEP 6 cm H2O and FiO2 up to 0.3 or persistent/worsening respiratory distress), mode of respiratory support was stepped up to nasal intermittent positive pressure ventilation (NIPPV) support. The NIPPV support was started on peak inspiratory pressure (PIP) of 14 cm H2O and escalated as required up to 20 cm H2O and 0.4 FiO2 to alleviate respiratory distress and maintain the target oxygen saturation. A trial of NIPPV support was opted before invasive ventilation, considering it to be a superior modality of respiratory support to CPAP for preterm neonates with respiratory distress (16). The following criteria were used for considering mechanical ventilation: (a) repeated episodes of apnea defined as more than four episodes of apnea per hour or more than two episodes requiring bag and mask ventilation, (b) hypoxia defined as FiO2 >0.40 to maintain SpO2, and (c) respiratory acidosis with PaCO2 >60 mmHg and pH <7.20. The same criteria were utilised for the requirement of respiratory support post-surfactant therapy.

On admission, as per the unit protocol, a baseline arterial blood gas and CXR were performed. Baseline lung ultrasound scoring was done and was denoted as “pre-surf LUS”. The surfactant was administered as per the European consensus guidelines (early rescue surfactant therapy if FiO2 >0.3 on CPAP pressure of at least 6 cm H2O) (9). The neonates requiring NIPPV or invasive ventilation were also given rescue surfactant therapy in case of FiO2 >0.3 and PEEP >6 cm H2O. All the neonates were administered surfactant via the intratracheal route and the INSURE (intubate-surfactant-extubate) technique was utilised for the neonates on non-invasive ventilation. The type of surfactant administered was as per the available and affordable option and hence, both beractant and poractant alfa were administered at a dose of 100 mg/kg of phospholipid. Following this, a repeat LUS was done after 6 h of the first dose of surfactant and was denoted as “post-surf LUS”. A second dose of surfactant at 100 mg/kg of phospholipid was administered if the FiO2 value remained above the cutoff (9). Apart from this, SpO2/FiO2 ratio and a/A ratio were calculated for all the neonates prior to surfactant therapy, and CXR grading was done based on the radiological characteristics, namely, reticulogranular pattern, radiolucency, cardiac silhouette, and air-bronchograms—Stage 1: fine granular pattern with few air-bronchograms; Stage 2: distinct granularity and excessive air-bronchograms; Stage 3: increased opacity with reduced air-bronchograms; and Stage 4: diffuse bilateral opacified lung (white-out) with lack of cardiac borders and loss of all air-bronchograms (17).

## Lung ultrasound scoring

Lung ultrasound was done on the Philips Affinity 50G (Koninklijke Philips, Nevada, USA) machine with the “hockey stick” L15–17 MHz transducer. Each lung was divided into three areas (upper anterior, lower anterior, and lateral) and scored.

**Figure 1:**
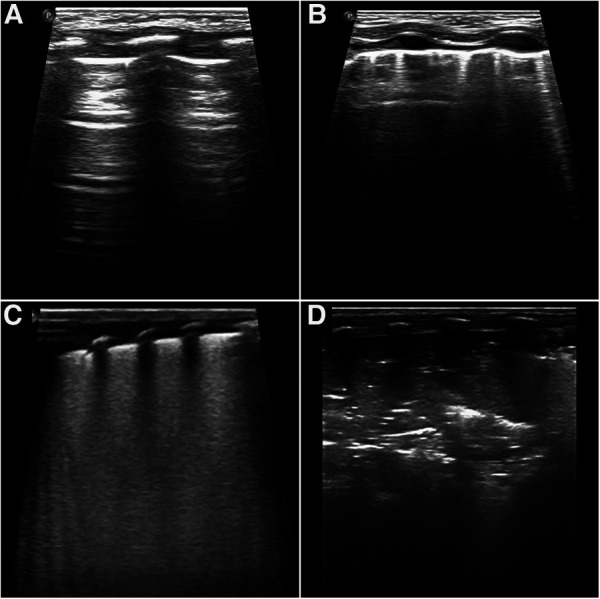
Score values correspond to different patterns as shown. Scores were given as follows: (**A**) 0, presence of only A-lines; (**B**) 1, presence of ≥3 well-spaced B lines; (**C**) 2, presence of crowded and coalescent B lines with or without consolidations limited to sub-pleural space; (**D**) 3, presence of extended consolidation.

Each area of the lung was given a score between 0 and 3, with the total score ranging from 0 to 18, where 0 indicated A-pattern [defined by the presence of A-lines only (horizontal, parallel echogenic lines under the pleural line)]; 1 indicated B-Pattern [defined as the presence of ≥3 well-spaced B lines (well defined vertical lines that originated on the pleural line, ran perpendicular to and obscured the A-lines)]; 2 indicated severe B pattern (defined as the presence of crowded and coalescent B lines with or without consolidation limited to subpleural space and/or white out of lung); and 3 indicated extended consolidation (Figure 1) (8).

Lung ultrasound was performed by a clinician who had received formal training under senior faculty and who had at least 6 months of experience in the NICU. The pre-recorded ultrasound videos were scored by a single trained blinded observer with an acceptable level of expertise.

Maternal characteristics (antenatal corticosteroid cover, mode of delivery, and risk factors) and neonatal characteristics [sex, gestational age, birth weight, weight for gestational age, the requirement of resuscitation at birth, age at enrolment, doses of surfactant, SpO2/FiO2 and a/A ratios just prior to surfactant administration and before each dose, CXR grading before each dose of surfactant, days on mechanical ventilation, days on non-invasive ventilation, duration of oxygen support (days), length of NICU stay (days), bronchopulmonary dysplasia (%)] were recorded. The neonates were subdivided as per respiratory pathology into RDS, transient tachypnea of the newborn (TTNB), and congenital pneumonia. The criteria for diagnosing congenital pneumonia was respiratory distress along with at least two laboratory parameters positive for sepsis (c-reactive protein ≥6 mg/L, thrombocytopenia with platelet count <100,000/mm^3^, immature:total neutrophil ratio >0.2:1, neutropenia as per newborn cutoffs) (18). RDS was diagnosed based on clinical respiratory distress, chest x-ray showing features consistent with the disease, and no laboratory features of sepsis (19). The presence of respiratory distress (transient and self-limiting) with a radiographic picture of inter-fissural fluid or interstitial fluid was diagnosed as TTNB (20).

## Statistics

Categorical variables were expressed as frequency (percentage) and compared using the chi-squared test or Fisher's exact test, as needed. Quantitative data was expressed as mean ± SD and median (IQR). A value of *p *< 0.05 was considered statistically significant. Receiver operating characteristic (ROC) analysis was used to evaluate the reliability of the LUS to predict the need for surfactant treatment and re-treatment; area under the curve (AUC) and reliability data were reported with confidence intervals (CIs). Paired analysis was done for comparison of LUS at 0 and 6 h post surfactant. Correlational analysis using Pearson's coefficient was done to determine the correlation of LUS with the SpO2/FiO2 ratio, a/A ratio, and CXR grading. To determine the correlation between LUS and CXR at <3 and >3 h of life at enrolment, correlational analysis was conducted using the Spearman coefficient.

The sample size was calculated using observational data from the previous year where surfactant was administered to approximately 50% of NICU-admitted infants who fulfilled the same inclusion criteria and followed the same surfactant administration protocol. To achieve an AUC of ≥0.7 in ROC analysis with *α* error of 0.05 and power of 0.95, 100 samples were needed.

## Results

**Figure 2:**
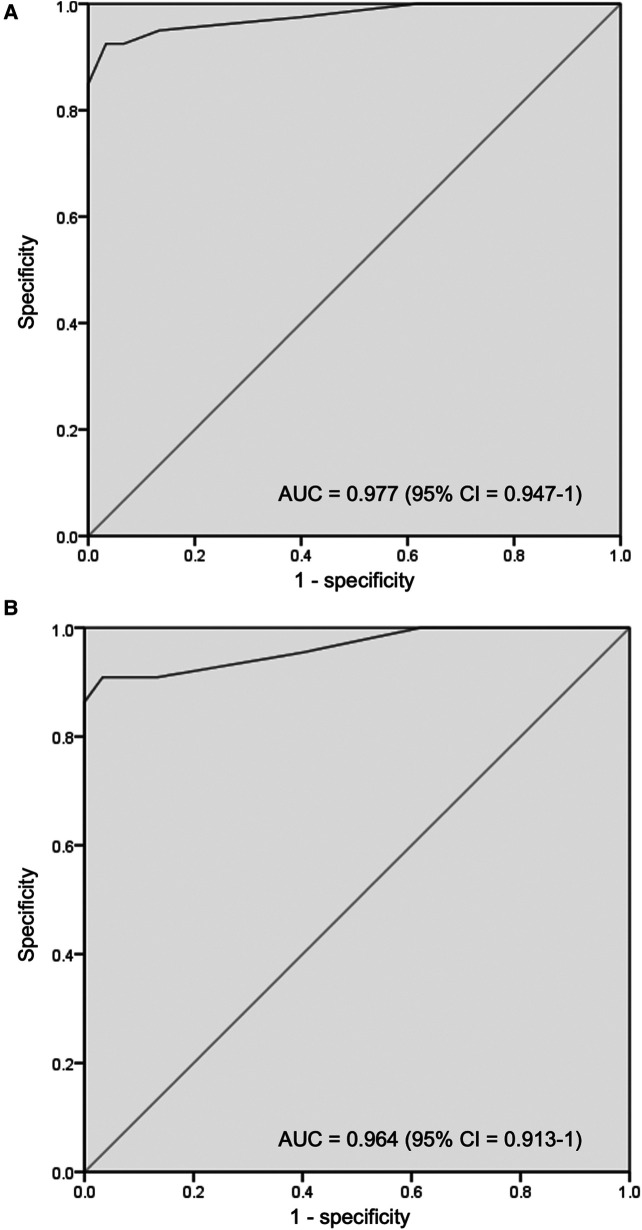
(**A**) Receiver operating characteristic (ROC) curve for pre-surfactant LUS cutoff for the requirement of surfactant therapy (**B**) receiver operating characteristic (ROC) curve for pre-surfactant LUS cutoff for the requirement of repeat surfactant therapy.

**Figure 3:**
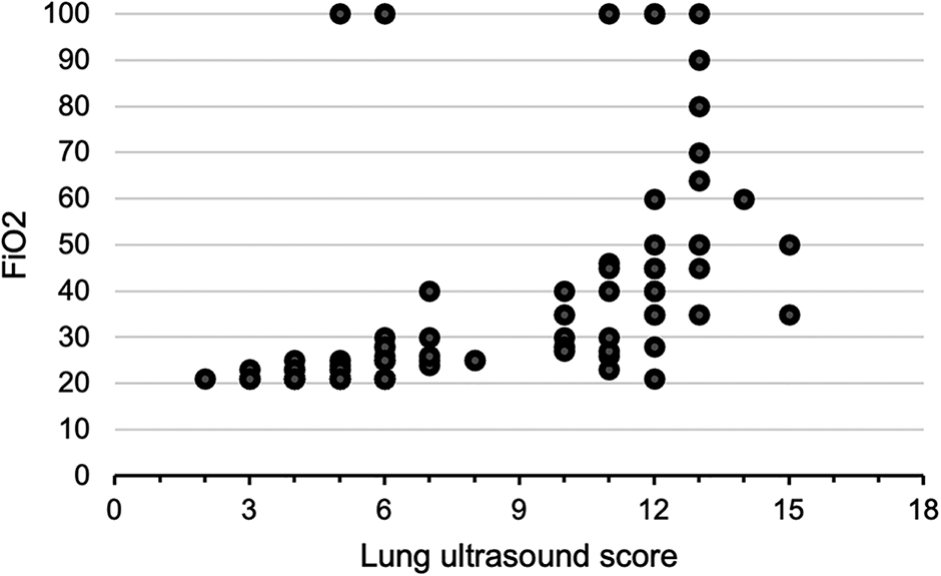
Scatter plot showing the distribution of pre-surfactant LUS with corresponding FiO2.

A total of 100 neonates were included in the study. The mean (SD) gestation of enrolled neonates was 31.06 ± 2.12 weeks and the mean birthweight was 1,412 ± 391 g. Upon diagnosis, 58% of the enrolled neonates had RDS, 30% had congenital pneumonia, and 12% had TTNB. Baseline characteristics of the study population are reported in Table 1. Out of the 100 enrolled patients, 40 neonates received surfactant therapy. Among these, 18 received beractant and 22 received poractant alfa surfactant preparation. The mean age at first dose of surfactant was 1.9 ± 1 h of life. The characteristics of the neonates who received surfactant therapy are given in Table 2. The pre-surf LUS was done at the hour of enrolment and with an AUC 0.977, 95% CI (0.947–1), and *P* < 0.001, the cutoff score as per ROC curve was 7 (sensitivity 92.5%, specificity 96.7%, positive predictive value (PPV) 94.87%, and negative predictive value (NPV) 95.08%) (Figure 2A and Table 3). The mean age of the neonates at the time of pre-Surf LUS was 1.37 ± 0.84 h. The mean LUS of neonates with RDS, congenital pneumonia, and TTNB were 8.9 ± 3.5, 8.6 ± 3.7, and 4.8 ± 1.6, respectively. The mean (SD) LUS for neonates with RDS who did not require surfactant was 5.5 ± 1.6 and the median (IQR) was 4 (4–6), and the mean LUS for neonates with RDS who required surfactant was 11.6 ± 2 and the median was 12 (11–13). This result was statistically significant with *P* < 0.0001. The pre-surf LUS cutoff determining requirement of >1 dose of surfactant was 10 (sensitivity 100%, specificity 86.36%, PPV 95.24%, and NPV 100%) as per ROC with an AUC 0.964, 95% CI (0.913–1), and *P* < 0.001 (Figure 2B). A total of 23 neonates required a repeat dose of surfactant, 17 of these were diagnosed with RDS and the remaining 6 had congenital pneumonia. The mean (SD) age at repeat dose of surfactant therapy was 7.5 ± 0.8 h of life. In neonates requiring one dose of surfactant therapy, the LUS decreased by a median value of 3.24 (2.44–4.05) over 6 h. The mean difference between pre- and post-surf LUS for neonates who received beractant was 3.73 ± 1.35 (*P* 0.1) and for neonates who received poractant alfa, it was 2.94 ± 1.76 (*P* 0.12). Figure 3 shows the distribution of LUS values with the corresponding FiO2. A correlation of −0.75 (*P* < 0.001) was found between pre-surf LUS and SpO2/FiO2 ratio and a correlation of −0.235 (*P* 0.144) was found between post-surf LUS and SpO2/FiO2 ratio after 6 h of surfactant. Between pre-surf LUS and a/A, the correlation was −0.65 (*P* < 0.001), and between post-surf LUS and a/A at 6 h post-therapy, it was −0.075 (*P* 0.645). A significant correlation was found between pre-surf LUS and first CXR at 0.801 (*P* < 0.001) and post-surf LUS and CXR in babies requiring a repeat dose of surfactant at 0.811 (*P* < 0.001). The correlation between pre-surf LUS and CXR at <3 h of enrolment was 0.829 (*P* < 0.001) and it was 0.832 (*P* < 0.001) at ≥3 h of enrolment. A total of 80 neonates were followed up till discharge. Six neonates among the enrolled patients died. Caregivers of 12 and 2 neonates opted to leave against medical advice due to financial constraints and poor neurodevelopmental outcomes (secondary to post-hemorrhagic hydrocephalus), respectively. A total of 80 neonates were given non-invasive respiratory support after birth, 31 of which required escalation to mechanical ventilation, and 22 of these were administered surfactant therapy as per eligibility criteria.

**Table 1:** Characteristics of the study cohort, *n* = 100.

Maternal characteristics (*n* = 100)	*N* (%)
Maternal history	
Preterm labor	49 (49)
Severe pre-eclampsia/eclampsia	21 (21)
Antepartum haemorrhage	10 (10)
Antenatal Doppler changes	16 (16)
Severe oligo/anhydramnios	2 (2)
Fetal bradycardia	1 (1)
Scar tenderness	1 (1)
Antenatal steroid course	
Complete	42 (42)
Incomplete	58 (58)
Mode of delivery	
Caesarean section	77 (77)
Vaginal delivery	23 (23)
Neonatal characteristics (*n* = 100)	*N* (%)
Male	50 (50)
Inborn	80 (80)
Gestational age (weeks)	
<28	9 (9)
28–31 + 6	48 (48)
32–33 + 6	43 (43)
Birth weight (g)	
<1,000	16 (16)
1,000–1,499	45 (45)
≥1,500	39 (39)
Small for gestational age	21 (21)
Hours of life at enrolment	
<3	86 (86)
≥3	14 (14)
Resuscitation at birth	
No resuscitation	46 (46)
Physical stimulation	5 (5)
Positive pressure ventilation	29 (29)
Delivery room intubation	18 (18)
Chest compressions	2 (2)
Medications	–
Respiratory pathology	
Respiratory distress syndrome	58 (58)
Congenital pneumonia	30 (30)
Transient tachypnea of the newborn	12 (12)
Surfactant therapy	40 (40)
Number of doses of surfactant required	
1	17 (43)
2	23 (58)
Type of surfactant used	
Beractant	22 (55)
Poractant alfa	18 (45)
LUS, median (IQR)	
1st dose of surfactant	6 (5–12)
2nd dose of surfactant	8.5 (6–10.5)
SpO2/FiO2 ratio, median (IQR)	
1st dose of surfactant	3.7 (2.3–4.5)
2nd dose of surfactant	4.5 (2.8–4.6)
a/A ratio, median (IQR)	
1st dose of surfactant	0.42 (0.28–0.55)
2nd dose of surfactant	0.345 (0.275–0.38)
Median LUS as per respiratory pathology	
Respiratory distress syndrome	10 (5–12)
Congenital pneumonia	7 (5.5–12)
Transient tachypnea of the newborn	5 (4–6)

LUS, lung ultrasound score; SpO2/FiO2, oxygen saturation to fraction of inspired oxygen ratio; a/A, arterial/Alveolar oxygen pressure.

**Table 2:** Characteristics of the neonates receiving surfactant therapy, *n* = 40.

Neonatal characteristics	*N* (%)
Male	23 (58)
Inborn	31 (78)
Mean gestational age (weeks)	30.1 ± 2.3
Mean birth weight (g)	1,344 ± 441
Respiratory pathology	
Respiratory distress syndrome	29 (73)
Congenital pneumonia	11 (27)
Transient tachypnea of the newborn	0 (0)
Hours of life at 1st dose of surfactant	
<3	28 (70)
≥3	12 (30)
Mean age at 1st dose of surfactant (hours)	
Inborn	1.6 ± 0.8
Outborn	2.9 ± 0.9

**Table 3:** Diagnostic utility of lung ultrasound score with surfactant therapy.

Lung ultrasound score	Surfactant therapy	
	Yes	No
≤7	3	58
>7	37	2
Sensitivity	92.5% (76.91–98.43	
Specificity	96.67% (88.47–99.59)	
Positive predictive value	94.87% (82.52–98.64)	
Negative predictive value	95.08% (86.67–98.29)	
Positive likelihood ratio	27.75 (7.08–108.71)	
Negative likelihood ratio	0.08 (0.03–0.23)	

## Discussion

In this study, a quantitative lung ultrasound score was observed to be an excellent predictor of the need for surfactant therapy in respiratory distress in preterm neonates. It was noted that this ultrasonographic marker of lung aeration has a significant correlation with other clinical markers (a/A ratio and SpO2/FiO2 ratio) and radiological markers (CXR grading).

In 2012, Raimondi et al. (21) highlighted the role of lung ultrasonography in respiratory distress in newborns and since then there have been multiple studies to evaluate the same. It is now increasingly being recognised as a primary modality of choice for the assessment of respiratory distress in newborns. However, the existing cutoffs for surfactant replacement therapy have been developed through studies from high-income countries enrolling neonates with RDS and limited data from the Indian population exist for the same, especially in the setting of congenital pneumonia.

Our study population was similar in terms of gestational age and birth weight to the studies previously reported (12, 13). The median time for the first LUS assessment in the present study was 1 h. Taking into account that the study population included newborns that were delivered in the hospital as well as those that were referred from outside hospitals for respiratory distress, this time of assessment was lower as compared to other studies. In their research, Perri et al. reported the time of first assessment as 3.3 (1.8) hours and 2.5 h (12, 13). It has been observed that LUS may vary and even worsen in the first 4 h of life owing to the liquid clearance from airways (22). Therefore, earlier evaluation within the first 1–2 h of life is expected to increase the clinical value of the score and reduce false positive results. Previously, the superiority of LUS done as early as 5–10 min of life has been reported (23).

Developing countries have a higher incidence of congenital pneumonia (24). Moreover, it is difficult to differentiate and establish this diagnosis at birth and the time frame for early rescue surfactant therapy is limited. For this reason, all neonates with respiratory distress were enrolled in our study cohort in contrast to previous studies where inclusion criteria for respiratory pathology were restricted to RDS. Notably, almost one-third of our study population was diagnosed with congenital pneumonia and the average LUS of these neonates was comparable with RDS. Therefore, through this study, the diagnostic utility of lung ultrasound for timely surfactant replacement therapy is highlighted in congenital pneumonia as well as RDS.

For an objective assessment of the requirement of surfactant in neonates with respiratory distress, Brat et al. (8) developed and established LUS through their study in 2015. However, the LUS cutoff as per their study in 65 infants <34 weeks gestation was 4 in comparison to our cutoff score of 7. The LUS cutoff for the second dose of surfactant as per our study was 10. Our findings were similar to those of De Martino et al. (14), who studied more preterm populations (≤30 weeks gestational age) but had ultrasound protocols that matched ours. In similar studies, Perri et al. reported the LUS cutoff for surfactant treatment to be 5 and for retreatment to be 7 (12, 13). A recent study by Raimondi et al. reported 9 as the LUS cutoff for surfactant therapy (11). Another recently published study (25) from India reported an optimal cutoff score ≥9 for giving surfactants. However, the sensitivity and specificity for the same was lower. Additionally, posterior chest areas were also included in their scoring system to calculate the final LUS. This may account for the differences in their findings as compared to ours.

In the present study, we found that LUS decreased after surfactant administration. This was expected owing to changes in lung mechanics after surfactant replacement, which has been reflected in some of the previous studies (11, 13).

In the present study, the LUS correlated significantly with SpO2/FiO2 ratio, a/A ratio, and CXR. Brat et al. (8) also showed a significant correlation between LUS and a/A ratio, and Perri et al. (12, 13) observed a significant correlation between LUS and SpO2/FiO2 ratio and LUS and CXR. Since the calculation of mean airway pressures was not possible in all neonates across the various modes of respiratory support used (especially CPAP), oxygenation indices considered for correlation with LUS were SpO2/FiO2 and a/A ratios. SpO2/FiO2 ratio was utilised in the study due to it being an accurate surrogate marker of oxygenation status (26). Raschetti et al., in their quality improvement project, noted FiO2 to be a later predictor of surfactant therapy as compared to LUS, and FiO2 based criteria was shown to have increased the duration of oxygen exposure due to delayed surfactant therapy (10). This affirms that LUS in conjunction with other baseline parameters is a better predictor for surfactant therapy in preterms as compared to FiO2 alone and with comparable findings on CXR.

Our study methodology was in accordance with the existing standard guidelines for surfactant use. The first LUS was done relatively earlier as compared to previous studies, therefore, the reliability is expected to be more when compared with other baseline parameters at enrolment with lower false positivity. The scoring of all lung ultrasounds by a single trained observer eliminated the risk of interobserver bias. The inclusion of all neonates with respiratory distress at birth foregrounded the wider applicability of LUS in pathologies other than RDS.

The study, however, was not devoid of limitations. It was conducted at a single centre with a relatively small sample size. The number of extremely preterm neonates was rather limited, therefore limiting the generalizability of the results for that population. Additionally, the role of LUS in late preterm neonates (gestational age >34 weeks) with respiratory distress remains to be studied. The LUS score was correlated with CXR findings for all neonates with respiratory distress, however, the grading scale used for the same was as per the radiological characteristics of RDS. The study was conducted in a low-resource setting and therefore, both preparations of surfactant (beractant and poractant alfa) were administered to the neonates according to availability and affordability. Furthermore, poractant alfa had to be administered at a dose lower than the recommended 200 mg/kg of phospholipid as it was a more expensive option. The mean airway pressure could not be recorded as part of the study due to variations in the mode of respiratory support and, therefore, the LUS could not be correlated against better oxygenation indices such as oxygenation index (OI) and oxygen saturation index (OSI).

## Conclusion

The study predicted an optimal LUS cutoff of 7 and 10 for the need for the first dose of surfactant and re-treatment, respectively, in neonates <34 weeks gestational age with respiratory distress. LUS correlated significantly with SpO2/FiO2 and a/A ratios and findings on CXR. However, larger multi-centric trials in India as well as low- and middle-income settings, including neonates >34 weeks gestational age with respiratory distress, are required to validate these cutoffs for surfactant replacement therapy. Future studies aimed at more homogeneity, with respect to the type of surfactant, at the recommended dose of phospholipids, and utilising superior oxygenation indices are warranted to further elaborate upon the clinical utility of lung ultrasound scoring.

## Data Availability

The raw data supporting the conclusions of this article will be made available by the authors, without undue reservation.

## References

[1] National Neonatal Perinatal Database 2002–03, NNPD Network. *Supported by Indian council of medical research, New Delhi, nodal center, AIIMS, New Delhi*. Available at: http://www.newbornwhocc.org/pdf/nnpd_2002-03.PDF

[2] Mathai SS Raju U Kanitkar M. Management of respiratory distress in the newborn. Med J Armed Forces India. (2007) 63(3):269–72. 10.1016/S0377-1237(07)80152-3 27408014 PMC4922755

[3] Bahadue FL Soll R. Early versus delayed selective surfactant treatment for neonatal respiratory distress syndrome. Cochrane Database Syst Rev. (2012) 11(11):CD001456. 10.1002/14651858.CD001456.pub2 23152207 PMC7057030

[4] Kurepa D Zaghloul N Watkins L Liu J. Neonatal lung ultrasound exam guidelines. J Perinatol. (2018) 38(1):11–22. 10.1038/jp.2017.140 29144490

[5] Bhoil R Ahluwalia A Chopra R Surya M Bhoil S. Signs and lines in lung ultrasound. J Ultrason. (2021) 21(86):e225–33. 10.15557/JoU.2021.0036 34540277 PMC8439137

[6] Bouhemad B Brisson H Le-Guen M Arbelot C Lu Q Rouby JJ. Bedside ultrasound assessment of positive end-expiratory pressure-induced lung recruitment. Am J Respir Crit Care Med. (2011) 183(3):341–7. 10.1164/rccm.201003-0369OC 20851923

[7] Via G Storti E Gulati G Neri L Mojoli F Braschi A. Lung ultrasound in the ICU: from diagnostic instrument to respiratory monitoring tool. Minerva Anestesiol. (2012) 78(11):1282–96. 22858877

[8] Brat R Yousef N Klifa R Reynaud S Shankar Aguilera S De Luca D. Lung ultrasonography score to evaluate oxygenation and surfactant need in neonates treated with continuous positive airway pressure. JAMA Pediatr. (2015) 169(8):e151797. 10.1001/jamapediatrics.2015.1797 26237465

[9] Sweet DG Carnielli V Greisen G Hallman M Ozek E Te Pas A European consensus guidelines on the management of respiratory distress syndrome—2019 update. Neonatology. (2019) 115(4):432–50. 10.1159/000499361 30974433 PMC6604659

[10] Raschetti R Yousef N Vigo G Marseglia G Centorrino R Ben-Ammar R Echography-guided surfactant therapy to improve timeliness of surfactant replacement: a quality improvement project. J Pediatr. (2019) 212:137–143.e1. 10.1016/j.jpeds.2019.04.020 31079857

[11] Raimondi F Migliaro F Corsini I Meneghin F Pierri L Salomè S Neonatal lung ultrasound and surfactant administration. Chest. (2021) 160(6):2178–86. 10.1016/j.chest.2021.06.076 34293317

[12] Perri A Riccardi R Iannotta R Di Molfetta DV Arena R Vento G Lung ultrasonography score versus chest x-ray score to predict surfactant administration in newborns with respiratory distress syndrome. Pediatr Pulmonol. (2018) 53(9):1231–6. 10.1002/ppul.24076 29870158

[13] Perri A Tana M Riccardi R Iannotta R Giordano L Rubortone S Neonatal lung ultrasonography score after surfactant in preterm infants: a prospective observational study. Pediatr Pulmonol. (2019) 55(1):116–21. 10.1002/ppul.24566 31710177

[14] De Martino L Yousef N Ben-Ammar R Raimondi F Shankar-Aguilera S De Luca D. Lung ultrasound score predicts surfactant need in extremely preterm neonates. Pediatrics. (2018) 142(3):e20180463. 10.1542/peds.2018-0463 30108142

[15] Kartikeswar G Parikh T Pandya D Pandit A. Lung ultrasound (LUS) in pre-term neonates with respiratory distress: a prospective observational study. Lung India. (2022) 39(5):417. 10.4103/lungindia.lungindia_13_22 36629201 PMC9623872

[16] Lemyre B Laughon M Bose C Davis PG. Early nasal intermittent positive pressure ventilation (NIPPV) versus early nasal continuous positive airway pressure (NCPAP) for preterm infants. Cochrane Database Syst Rev. (2016) 12(12):CD005384. 10.1002/14651858.CD005384.pub2 27976361 PMC6463790

[17] Jain SN Modi T Varma RU. Decoding the neonatal chest radiograph: an insight into neonatal respiratory distress. Indian J Radiol Imaging. (2020) 30(4):482–92. 10.4103/ijri.IJRI_281_20 33737778 PMC7954172

[18] Mathur NB Garg K Kumar S. Respiratory distress in neonates with special reference to pneumonia. Indian Pediatr. (2002) 39(6):529–37. 12084946

[19] Reuter S Moser C Baack M. Respiratory distress in the newborn. Pediatr Rev. (2014) 35(10):417–28. 10.1542/pir.35.10.417 25274969 PMC4533247

[20] Guglani L Lakshminrusimha S Ryan RM. Transient tachypnea of the newborn. Pediatr Rev. (2008) 29(11):e59–65. 10.1542/pir.29.11.e59 18977854

[21] Raimondi F Migliaro F Sodano A Umbaldo A Romano A Vallone G Can neonatal lung ultrasound monitor fluid clearance and predict the need of respiratory support? Crit Care. (2012) 16(6):R220. 10.1186/cc11865 23151314 PMC3672599

[22] Blank DA Kamlin COF Rogerson SR Fox LM Lorenz L Kane SC Lung ultrasound immediately after birth to describe normal neonatal transition: an observational study. Arch Dis Child Fetal Neonatal Ed. (2018) 103(2):F157–62. 10.1136/archdischild-2017-312818 28659360

[23] Badurdeen S Kamlin COF Rogerson SR Kane SC Polglase GR Hooper SB Lung ultrasound during newborn resuscitation predicts the need for surfactant therapy in very- and extremely preterm infants. Resuscitation. (2021) 162:227–35. 10.1016/j.resuscitation.2021.01.025 33548362

[24] Duke T. Neonatal pneumonia in developing countries. Arch Dis Child Fetal Neonatal Ed. (2005) 90(3):F211–9. 10.1136/adc.2003.048108 15846010 PMC1721897

[25] Roy T Pal S Sardar S Mukherjee S Ghosh M. Prediction of surfactant requirement in Indian preterm infants by lung ultrasound scores: a diagnostic accuracy study from a developing country. Eur J Pediatr. (2023) 182(2):625–32. 10.1007/s00431-022-04717-z 36414868

[26] Khemani RG Patel NR Bart RD 3rd Newth CJL. Comparison of the pulse oximetric saturation/fraction of inspired oxygen ratio and the PaO2/fraction of inspired oxygen ratio in children. Chest. (2009) 135(3):662–8. 10.1378/chest.08-2239 19029434

